# Socioeconomic determinants of malnutrition among mothers in the Amoron’i Mania region of Madagascar: a cross-sectional study

**DOI:** 10.1186/s40795-018-0212-4

**Published:** 2018-02-17

**Authors:** Lantonirina Ravaoarisoa, Lalhyss Randriamanantsaina, Julio Rakotonirina, Jean de Dieu Marie Rakotomanga, Philippe Donnen, Michèle Wilmet Dramaix

**Affiliations:** 1 Institut National de Santé Publique et Communautaire, Antananarivo, Madagascar; 2 Faculté de Médecine d’Antananarivo, Antananarivo, Madagascar; 3 Ecole de Santé Publique de l’Université Libre de Bruxelles, Bruxelles, Belgium

**Keywords:** Nutritional status, Mother, Malnutrition, Socioeconomic determinant

## Abstract

**Background:**

Maternal malnutrition, which has been a problem in Madagascar for several years, has been rising despite interventions to improve the situation. This study aims to identify the socioeconomic determinants of malnutrition among mothers who are one of the most vulnerable groups.

**Methods:**

A cross sectional study was carried out among 670 mothers aged 18 to 45 living in the Amoron’i Mania region of Madagascar. The study was conducted during the post-harvest period. The nutritional status of mothers was assessed by anthropometry. A Body Mass Index (BMI) lower than 18.5 kg/m^2^ or an arm circumference lower than 220 mm were used to define malnutrition. Data on the characteristics of the mothers and their households were also collected. Multiple logistic regression was used to identify factors associated with maternal malnutrition.

**Results:**

The prevalence of maternal undernutrition is estimated at 17% (95% CI: 14–20) according to BMI and 9% (95% CI: 7–11) for Mid Upper Arm Circumference (MUAC). In the multivariate analysis, using BMI, the factors significantly associated with malnutrition were: the household size equal to or greater than 6 (AOR = 1.59 [1.04–3.42], *p* = 0.029) and use of unsafe water source (AOR = 1.99 [1.02–3.85], *p* = 0.030). For the MUAC, the factors associated are: use of unsafe water source (AOR = 2.82 [1.01–7.97], *p* = 0.041) and increased number of children under five years old (AOR = 1.38 [1.02–1.89], *p* = 0.025).

**Conclusion:**

This study confirmed the importance of mothers’ malnutrition in the study area. Fight against maternal malnutrition needs interventions to improve access to safe drinking water and to promote family planning.

## Background

Mothers are a group that is vulnerable to nutritional disorders because of the specific needs related to reproduction [1]. A child’s development depends entirely on the mother’s nutritional status during fetal life and the first six months of life [2]. Current knowledge shows the adverse effects of maternal malnutrition on their health and that of their children [3].

Maternal malnutrition is a concern in several countries. In many South Asian and sub-Saharan African countries, more than 20% of women are undernourished (Body Mass Index or BMI < 18.5). In some of those countries, it can affect up to 40% of women [4]. In many countries in South-Central Asia, more than 10% of women are less than 145 cm in height [4]. Furthermore, 30% of women of reproductive age worldwide have anemia [5]. To address maternal malnutrition, many actions with evidence of effectiveness have been identified such as iron folate and multiple micronutrients supplementations, deworming, iodisation of salt,… [6–8].

Madagascar is among the countries heavily affected by maternal malnutrition [9, 10]. In 2009, a high prevalence of undernutrition (BMI < 18.5) among women of reproductive age was reported (26.7%) and 38.3% of pregnant women had anemia [11]. A high proportion of pregnant women have low weight gain during pregnancy (76% to 100%). Poor nutritional status before conception is an important determining factor of this low weight gain [12, 13].

Poor maternal nutritional status has been a rising trend in Madagascar for several years [11]. Strategies chosen to solve this issue at the national level remain limited and nonspecific. Available data have provided situational awareness but little information to explain the causes. The present study was conducted to identify the socioeconomic determining factors of maternal malnutrition in the region Amoron’ Mania. It focused on rural areas where malnutrition issues are much more prevalent [11]. Knowing these factors will help readjust strategies to fight maternal malnutrition.

## Methods

### Study site

The study was conducted in Amoron’i Mania, one of Madagascar’s 22 regions. The region comprises 4 health districts, 53 communes, of which 52 are located in rural areas, and about 580,000 inhabitants. This region has the highest undernutrition (BMI < 18.5) prevalence among women of reproductive age in Madagascar. It was estimated at 41.6% in the Demographic Health Survey (DHS) results in 2008–2009 [11].

### Study population

A cross-sectional study was carried out. The study population included non-pregnant mothers between 18 and 45 years of age who had given birth more than 6 months earlier. Mothers under 18 years old were not included because of the difficulty in getting the guardian’s consent. Mothers over 45 years old were not included so as to only take into account mothers in their reproductive period, knowing that the fertility rate is very low among woman aged between 45 and 49 years old [10]. To ensure the validity of the weight measurements, we also excluded women who had given birth within the last 6 months. Indeed, there is a gradual reduction in pregnancy weight gain and stabilization in mothers’ weight around sixth months after delivery [14, 15].

### Sampling

A two-stage cluster sampling was used. The first stage aimed at selecting 30 “fokontany” (smallest administrative structure) out of the 760 in the region. It was done by systematic random sampling. The second stage was used to select, for each “fokontany”, eligible mothers from a list established by community workers. This was done by simple random sampling. The sample size was calculated on the basis of maternal undernutrition national prevalence (27%), 5% margin of error, 95% confidence level and a design effect of 2 [16]. The sample size was estimated at 606. Twenty-one subjects per cluster therefore had to be included. During data collection, 670 women were actually interviewed.

### Data collection

Data collection was conducted in July and August 2015, during the post-harvest period in the region. Nutritional status was assessed by anthropometric measurements such as weight, height and Mid Upper Arm Circumference (MUAC). Women were weighed with 100 g accuracy with a SECA electronic scale. Height was measured with 1 cm accuracy with a SECA wall mounted height rod. MUAC was measured on the left arm, midway between the acromion and the olecranon, with 1 mm accuracy with an adult-specific measuring tape.

Information about dietary, socioeconomic, health and reproductive characteristics were collected by interviewers. Regarding the mothers’ dietary practice, a 24-h recall was used [17, 18]. Interviewers asked and established the list of all foods taken by the mother the day before the survey. To minimize omissions, they focused on food intake based on the pre-established list to assess women’s dietary diversity. Information about the place and the people with whom the mother took the meal as well as the occurrence of an unusual event the day before the survey was collected to detect unusual food consumption [17] . The recall took into account any day of the week except for days with unusual consumption such as festive days or stays away from the household. Mother’s age, education level (last school year taken into account, primary education takes six years in Madagascar), occupation, marital status and husband’s occupation were collected. Information about gravidity, parity, number of children aged less than 5 years old, breastfeeding status and birth interval were collected.

Interviewers were recruited locally. Data collectors received training according to their mission. The investigator, a technician from the Nutrition Department of the Ministry of Public Health, and the regional nutrition manager supervised the data collection. The study was approved by the Malagasy Ministry of Health’s Ethics Committee.

### Variables

#### Nutritional status

The Body Mass Index (BMI) was calculated by dividing weight in kilograms by height in square meters. WHO defined standards were used to identify undernourished women, i.e. BMI below 18.5 kg/m^2^, height below 145 cm, and MUAC below 220 mm [14].

#### Dietary diversity

The dietary diversity score was calculated using the 10 categorized food groups according to the latest recommended Women’s Dietary Diversity Score [18]. The consumption of one or more foods in one group was worth 1 point and the maximum score was 10.

#### Social profile

The birth interval was calculated for the last two deliveries within the last five years. Afterwards, that interval was grouped using a 24-month threshold. Mothers who did not have two childbirths within the last five years and three primiparous women were classified in the 24 months or more group.

Household-related variables were studied, i.e. the household head sex, the quality of the water source used for meal preparation, the toilet type, the fuel used and the house type. The water source is considered safe if it comes from standpipes, public faucets, covered or protected wells, wells or boreholes with pumps and faucets inside or outside the dwelling.

#### Economic profile

Three indicators of economic profile were created considering the possession of household goods. We used the DHS Madagascar list to establish our list of goods. The first indicator refers to possession of movable property (furniture, radio, TV, telephone, bicycle, etc.), the second refers to possession of farming equipment and the third to possession of farm animals. The corresponding scores for these properties were established by principal component analysis (PCA). The scores were categorized into three groups (high, medium and low) based on possible values close to the tertiles. The period (number of months) in which a household consumes its annual rice production was also collected. Rice production is considered as an important element of food security in the study area and can reflect the economic level of households. Rice is a Malagasy staple food and rice cultivation remains the main activity of most farming households in the region studied [19].

### Data analysis

Stata / IC 13.1 (StataCorp LP, College Station, USA) software was used to analyze the data. In bivariate analysis using logistic regression, the association between maternal undernutrition and other variables was estimated by the Odds Ratio (OR) with its confidence interval. The chi-square or chi-square for trend tests were used. Variables with *p*-value < 0.20 in bivariate analysis were considered for inclusion in a logistic regression model. A stepwise backward method was used for selection of statistically significant covariates. Categorical variables with more than two categories were transformed into indicators. The backward procedure used to select variables in the final model was based on the likelihood ratio. The adequacy of the final model was checked using Hosmer Lemeshow test. The adjusted ORs and their 95% confidence intervals were computed from the final multivariate logistic model. The significance level (*p*-value) was set at 0.05.

## Results

### Sample description

Table 1 shows the social characteristics of the 670 mothers included in the study. Nearly 3 out of 4 (73%) of them were illiterate or had not gone further than primary education. Agriculture was the main activity of the majority of mothers and 78% lived in couples. The dietary diversity score ranged from 1 to 7 and 88% of mothers had a score below 5. Age at the first pregnancy ranged from 12 to 34 years and 31% of the mothers had their first pregnancy before 18 years of age. Parity ranged from 1 to 17 and 73% of mothers had at least one child under 5 years old.

**Table 1:** Description of mothers according to their characteristics

Mothers’ characteristics (*n* = 670)	n	%
Age (year)		33 (7) ^a^
Education level		
Illiterate	94	14.0
Primary	393	58.7
Secondary 1st cycle	150	22.4
Secondary 2nd cycle	33	4.9
Occupation		
Farmer	605	90.3
Others	65	9.7
Marital status		
Couple	520	77.6
Single	150	22.4
Occupation of husband (*n* = 520)		
Farmer	428	82.3
Others	92	17.7
Dietary diversity score		3 (2–4) ^b^
Age at first pregnancy		19 (3) ^a^
≤ 15	59	8.8
16–17	148	22.1
≥ 18	463	69.1
Gravidity		4 (3–6) ^b^
Parity		4 (3–6) ^b^
Breastfeeding		
Yes	283	42.2
No	387	57.8
Age of last child (years)		3 (1–5) ^b^
Birth interval (months)		
< 24	95	14.2
≥ 24	575	85.8
Number of children < 5 years		
0	182	27.2
1	379	40.3
2	184	27.5
3 and +	33	5.1

^a^ Mean (SD)

^b^ Median (25%–75%)

Concerning the household characteristics, the median of household size was 6 and 81.2% of households were headed by men. Wood was the main fuel used for cooking (95.1%). Eighty-three percent of households drew water from unsafe sources (unprotected wells, unimproved wells, and rivers) and 26% had no toilet. Ninety-two percent of households owned the house they live in. In most cases, house walls were made of rammed earth (70.6%) with straw roofs (75.2%). As for rice production, it covered a median period of 4 months per year. In case of shortage, households bought or replaced rice with tubers (cassava, sweet potatoes).

### Mothers’ nutritional status

Table 2 describes the mothers’ nutritional status. Less than 10% of mothers had a height below 145 cm or a MUAC below 220 mm. According to BMI, 17% of mothers were undernourished (BMI < 18.5), 5.8% were overweight (25 ≤ BMI < 30) and 1.2% were obese (BM ≥ 30). Moderate (16 ≤ BMI ≤ 16.9) and severe (BMI < 16) malnutrition affected 2.8% and 0.7% of mothers respectively.

**Table 2:** Nutritional status of the mothers

Nutritional status (*n* = 670)	Mean [95% CI]	% [95% CI]
Weight (kg)	48.1 [47.5–48.6]	
Height (cm)	151.9 [151.4–152.3]	
< 145		8.8 [6.9–11.2]
MUAC (mm)	250.5 [248.7–252.3]	
< 220		8.7 [6.7–11.0]
BMI (kg/m^2^)	20.8 [20.6–21.0]	
< 18.5		17.0 [14.3–20.1]
18.5–24.9		76.0 [72.6–79.1]
25–29.9		5.8 [4.3–7.9]
≥ 30		1.2 [0.6–2.4]

*CI* confidence interval, *MUAC* Mid Upper Arm Circumference, *BMI* Body Mass Index

### Determinants of mothers’ nutritional status

Table 3 summarizes the results of the bivariate analysis that looked at the association between maternal undernutrition and socioeconomic factors. Using a BMI of 18.5 as a cutoff, malnourished was significantly more common among mothers who had household size equal to or greater than 6 (OR = 1.67 [1.10–2.53]), or used unsafe water source (OR = 2.13 [1.10–4.11]) and decreased when rice production availability duration increased (OR = 0.92 [0.85–0.99]). For MUAC, malnourished was significantly high among mothers who used unsafe water source (OR = 2.96 [1.04–8.34]), or had low movable property score (OR = 2.19 [1.10–4.40]) and among breastfeeding mother (OR = 1.77 [1.02–3.04]). Furthermore, undernutrition increased significantly as the education level decreased (*p* = 0.022) and as the number of children under five years old increased (OR = 1.41 [1.04–1.91]).

**Table 3:** Association of undernutrition with the characteristics of the mother and their household

	n	BMI < 18,5 (%)	OR (95% CI)	p^a^	MUAC < 220 mm (%)	OR (95% CI)	p^a^
Age (year)				0.087^b^			0.418
18–29	206	14.6	1		8.7	1	
30–39	322	16.5	1.15 (0.71–1.88)		7.5	0.84 (0.44–1.59)	
40–45	142	21.3	1.64 (0.94–2.86)		11.3	1.33 (0.65–2.70)	
Occupation				0.475			0.771
Farmer	605	17.4	1		8.8	1	
Others	65	13.8	0.77 (0.37–1.60)		7.7	0.87 (0.33–2.26)	
Marital status				0.226			0.327
Couple	520	17.9	1		9.2	1	
Single	150	14.0	0.75 (0.45–1.25)		6.7	0.70 (0.39–1.42)	
Education level				0.766			0.022
Illiterate & Primary	487	17.7	1.20 (0.45–3.20)		9.7	1.53 (0.72–3.32)	
Secondary 1st cycle	150	15.3	1.01 (0.35–2.90)		6.7	1	
Secondary 2nd cycle	33	15.2	1		0.0	NA	
Dietary diversity score				0.125			0.646
≤ 4	558	17.9	1		8.8	1	
5 and +	82	11.0	1.76 (0.85–3.64)		7.3	1.23 (0.51–2.96)	
Parity				0.554			0.353
0–3	293	16.0	1		7.5	1	
4 and +	377	17.8	1.13 (0.75–1.70)		9.6	1.30 (0.75–2.62)	
Breastfeeding				0.248			0.037
Yes	283	15.2	0.79 (0.53–1.21)		11.3	1.77 (1.02–3.04)	
No	387	18.4	1		6.7	1	
Birth interval (months)				0.732			0.277
≤ 24	95	15.8	1		11.6	1	
> 24	575	17.2	1.11 (0.61–2.00)		8.2	0.68 (0.34–1.36)	
Number of children < 5 years	670		0.88 (0.69–1.11)	0.280		1.41 (1.04–1.91)	0.029
Household size				0.016			0.292
≤ 5	310	13.2	1		7.4	1	
6 and +	360	20.3	1.67 (1.10–2.53)		9.7	1.34 (0.76–2.33)	
Water source				0.024			0.040
Safe	114	9.7	1		3.5	1	
Unsafe	556	18.5	2.13 (1.10–4.11)		9.7	2.96 (1.04–8.34)	
Toilet				0.895			0.526
Latrines	497	16.9	1		8.3	1	
No toilet	173	17.3	1.3 (0.65–1.63)		9.8	1.21 (0.67–2.19)	
Movable property score				0.578			0.022 ^b^
Low	218	15.1	0.87 (0.52–1.45)		11.9	2.19 (1.10–4.40)	
Medium	228	18.9	1.14 (0.70–1.84)		8.3	1.46 (0.71–3.06)	
High	224	17.0	1		5.8	1	
Farming equipment score				0.086 ^b^			0.606 ^b^
Low	351	17.9	1.20 (0.61–2.21)		9.7	1.38 (0.59–3.21)	
Medium	222	16.2	1.06 (0.55–2.04)		7.7	1.07 (0.42–2.66)	
High	97	15.5	1		7.2	1	
Farm animals score				0.489			0.926
Low	254	16.1	0.80 (0.50–1.28)		8.7	1.07 (0.65–2.05)	
Medium	195	15.4	0.75 (0.45–1.26)		9.2	1.14 (0.57–2.27)	
High	221	19.5	1		8.1	1	
Rice availability (month)	586		0.92 (0.85–0.99)	0.046	586	0.97 (0.88–1.08)	0.629

*BMI* Body Mass Index, *OR* Odds Ratio, *CI* Confidence interval, *MUAC* Mid Upper Arm Circumference, *NA* Not Applicable

^a^ Pearson’s chi square unless otherwise stated

^b^ Chi square for trend

Table 4 shows the results of the multivariate analysis. Using a BMI of 18.5 as a cutoff, household size and water source were significantly associated with maternal undernutrition. Adjusting for water source, mothers who have household size equal to or greater than 6 were more likely to be malnourished (AOR = 1.59 [1.04–3.42]). Adjusting for household size, those who used unsafe water source were more likely to be malnourished (AOR = 1.99 [1.02–3.85]). Concerning MUAC, 2 variables remained in the model: water source and number of children under five years old. Mothers who used unsafe water source were more likely to be malnourished than those who used safe water source (AOR = 2.82 [1.01–7.97]) and risk of undernutrition increased as the number of children under five years old increased (AOR = 1.38 [1.02–1.89]).

**Table 4:** Adjusted OR of undernutrition according to mother and household characteristics (*n* = 670)

	BMI < 18.5 kg/m^2^		MUAC < 220 mm	
Variable	AOR (95% CI)	p^a^	AOR (95% CI)	p^a^
Household size		0.029		
≤ 5	1			
6 and +	1.59 (1.04–3.42)			
Water source		0.030		0.041
Safe	1		1	
Unsafe	1.99 (1.02–3.85)		2.82 (1.01–7.97)	
Nb children < 5 years			1.38 (1.02–1.89)	0.025
Not included in the model because not significant	Age (p^b^=0.424) Dietary diversity score (p^b^=0.311) Farming equipment score (p^b^=0.759)		Education level (p^b^=0.822) Breastfeeding (p^b^=0.294) Movable property score (p^b^=0.400)	

*BMI* Body Mass Index, *MUAC* Mid Upper Arm Circumference, *AOR* adjusted Odds Ratio, *CI* Confidence interval

^a^ Likelihood ratio test

^b^ Likelihood ratio test when eliminated from the model

For the BMI model, if the duration of rice production availability was added, out of the 586 remaining subjects, household size kept the same effect, water source became non-significant, and the added variable had an effect at the limit of significance (AOR = 0.92 [0.84–1.00], *p* = 0.050).

## Discussion

Using BMI, the prevalence of undernutrition was estimated at 17%. This result is better than the latest available data (DHS 2008–2009), which showed a prevalence of undernutrition (BMI < 18.5) among women of 26.7% for the whole country and 41.6% for the Amoron’i Mania region [11]. The DHS included all women of reproductive age (15–49 years) and was conducted during the lean period. Our study, on the other hand, concerned the 18 to 45 age group, including women with at least one child over 6 months, and was conducted during the post-harvest period. But both studies used the same sampling procedure. The variation in nutritional status as a function of season is reported in numerous studies, mostly in populations living off agriculture. Nutritional status is much better in the harvest period than in the lean season in developing countries [19–21]. This may explain why we observed a lower prevalence of undernutrition in our study. Results from the DHS 2008–2009 showed that women under the age of 20 and over 40 have a slightly higher prevalence of undernutrition than other women (28% versus 25%) [11]. Our inclusion criterion in terms of age (18–45 years) probably selected women with less risk of undernutrition. Even with these methodological differences compared to the DHS, the decrease in the prevalence of malnutrition (41.6% to 17%) in 6 years (2009 to 2015) is remarkable. However, this study was carried out during a post-crisis period (political and socio-economic crisis) in which the population was in an unfavorable socio-economic situation. Indeed, 71.5% of the population was living below the national poverty line in 2013 [19]. It would be interesting to evaluate the interventions to fight malnutrition in the region that could explain the reduction in the prevalence of malnutrition.

Regarding the factors associated to the nutritional status of mothers, the household water source was kept in the BMI and MUAC models. In addition, the household size was included in the BMI model and the number of children under five years old in the MUAC model. Regarding household water sources, the link between a safe source and a good nutritional status of mothers stressed the importance of hygiene for health. Water quality is an essential element of hygiene and the drinking water supply is one of the most effective interventions to combat malnutrition [6, 22, 23]. The source of water is considered safe if it comes from fountains, public faucets, covered or protected wells, wells or boreholes equipped with pumps and faucets installed inside or outside the dwelling. In the study area, most households (83%) use water from unsafe sources including unprotected wells, rivers and unimproved sources. Mothers in these households are more malnourished than the mothers whose water source is safe. The use of non-potable water promotes the development of infectious diseases such as intestinal parasites and diarrheal diseases of microbial or viral origin [24, 25]. These diseases, frequently encountered in low-income countries, cause disturbances in the digestive system and directly affect its function. They are known as a direct cause of undernutrition [26].

As for the reproductive characteristics of mothers, the increase of the number of children under 5 increased malnutrition (AOR = 1.38 [1.02–1.89]. The number of children under five reflects many factors that characterize the reproductive life of mother, such as the workload for child care, breastfeeding, and birth interval. It is an indicator that could summarize the reproductive path of a mother during the last 5 years. A mother with 3 children under the age of 5 will have a larger workload and will not have time to recover physically because of the close succession of pregnancies and lactation [27]. Madagascar is making a great deal of effort to promote family planning, but more emphasis should be placed on birth spacing in light of the observed results for the number of children under five. In low-resource countries, the reproductive profile of mothers has a significant impact on their nutritional status and has been identified as a cause of undernutrition in the sub-Saharan region where Madagascar is located [28].

Mothers who have household size equal to or greater than 6 were more likely to be malnourished (AOR = 1.59 [1.04–3.42]). These households have more difficulty accessing food because of the important quantity of food needed to feed the whole family. Malnutrition also decreased when the availability of annual rice production increased. The latter may reflect the level of food security of households in this context where rice cultivation and consumption occupies a prominent place [19]. A low socioeconomic level is a known determinant of undernutrition, but it does not directly affect nutritional status. Its involvement is linked to the ability and capacity of women with a better socio-economic status to eat well, create a healthy environment for the family and have better health [29]. Studies carried out in the world have identified other components of socioeconomic status that expose mothers to undernutrition such as a low level of education, early pregnancy, close pregnancy, high parity, and unmet specific needs in the event of breastfeeding [30–33]. The importance of each component may be different from one context to another and it must guide response interventions based on evidence. For the study area, fight against maternal malnutrition needs interventions to improve access to safe drinking water and to promote family planning.

The two factors directly affecting nutritional status are diet and health status. Dietary diversity score was not associated with malnutrition. The dietary diversity score is an indicator of food quality but is also considered an indicator of household food security [34]. A more diverse diet is associated with adequate levels of calories and protein and better satisfaction of nutrient requirements [34, 35]. In this study, food information was limited to qualitative data. Quantitative assessment of energy and micronutrient intake is needed to complete the analysis on diet and nutritional status. This assessment should be carried out with that of physical activity, which is a major factor in the variation of energy requirements. Women farmers have physical activities involving high energy expenditure and this is the case for the population of this study [36]. Health status varies with time and its influence on nutritional status does not occur in a static way. Hence, the difficulty of establishing an indicator to really assess the health status of a woman, especially in the context of this study, where the means for diagnosing the diseases remain very limited.

## Conclusions

The problem of undernutrition affects mothers in the Amoron’i Mania region with considerable frequency. The study identified specific elements of the known socioeconomic determinants of malnutrition such as water hygiene, number of children under 5 years of age and household size. Although the determinants studied are not exhaustive, the information obtained will highlight some important interventions such as drinking water supply and birth spacing. In order to resolve the problem of malnutrition in this region, analyzing the adequacy between the strategies adopted and the real causes is necessary to readjust the interventions.

## Abbreviations

BMI: Body Mass Index
DHS: Demographic and Health Survey
MUAC: Mid Upper Arm Circumference
AOR: Adjusted Odds Ratio
PCA: Principal Component Analysis

## Authors’ information

Lantonirina R: Medical doctor, Master in Public Health, PhD student in Public health, assistant researcher at the “Institut National de Santé Publique et communautaire, Antananarivo, Madagascar” and at the faculty of Médicine of the University of Antananarivo Madagascar.

Lalhyss R: Master in community nutrition, team of the Nutrition Department of the Ministry of Health Madagascar.

JR: Medical doctor, PhD in Public health, Professor at the faculty of Médicine of the University of Antananarivo Madagascar and at the “Institut National de Santé Publique et communautaire, Antananarivo, Madagascar”.

JDMR: Medical Doctor, Associate Professor in Public health, Professor at the faculty of Médicine of the University of Antananarivo Madagascar and at the “Institut National de Santé Publique et communautaire, Antananarivo, Madagascar”.

DP: Medical doctor, PhD in Public health, Professor - Center of Research in Health Policy and Systems-International Health, School of Public Health, Université Libre de Bruxelles.

MWD: Master in Science (Physics), PhD in Public health, Professor - Center for Epidemiology, Biostatistics and Clinical Research, School of Public Health, Université Libre de Bruxelles.
